# EEG Correlation Coefficient Change with Motor Task Activation Can Be a Predictor of Functional Recovery after Hemiparetic Stroke

**DOI:** 10.3390/neurolint14030062

**Published:** 2022-09-19

**Authors:** Fei Zheng, Shin Sato, Kenji Mamada, Naoto Ozaki, Jin Kubo, Wataru Kakuda

**Affiliations:** 1 Graduate School of Medicine, International University of Health and Welfare, Tokyo 107-8402, Japan; 2 Department of Rehabilitation Medicine, International University of Health and Welfare Ichikawa Hospital, Chiba 272-0827, Japan; 3 Department of Clinical Laboratory Medicine, International University of Health and Welfare Ichikawa Hospital, Chiba 272-0827, Japan; 4 Department of Rehabilitation Medicine, International University of Health and Welfare School of Medicine, Chiba 286-8686, Japan

**Keywords:** stroke, hemiparesis, neural connection, electroencephalography, correlation analysis, motor task activation

## Abstract

Background: Recently, it was reported that the extent of cortico-cortical functional connections can be estimated by the correlation coefficient based on electroencephalography (EEG) monitoring. We aimed to investigate whether the EEG correlation coefficient change with motor task activation can predict the functional outcomes of hemiparetic stroke patients. Methods: Sixteen post-stroke hemiparetic patients admitted to our rehabilitation ward were studied. On admission, EEG recording to calculate the correlation coefficient was performed at rest and during motor task activation. For the analysis of the EEG data, the program software FOCUS (NIHON KOHDEN, Japan) was used. The motor function of paretic limbs was evaluated with the Fugl–Meyer Assessment (FMA) on admission and 4 weeks after admission. Results: Significant increases in the correlation coefficient with motor task activation were noted in C3-F3 or C4-F4, C3-F7 or C4-F8, and F3-F7 or F4-F8 of the lesional hemisphere. Among them, the rate of the correlation coefficient change in F3-F7 or F4-F8 in the lesional hemisphere was significantly correlated with the rate of the upper-limb FMA score change. Conclusion: The extent of the EEG correlation coefficient change with motor task activation in F3-F7 or F4-F8 of the lesional hemisphere may help predict the motor functional outcomes of hemiparetic upper limbs after stroke.

## 1. Introduction

In Japan, stroke is still one of the most common diseases, in spite of the development of preventive interventions for that. A total of approximately 290 patients develop stroke every year [1]. Stroke causes various types of neurological symptoms. Among them, hemiparesis is one of the most important symptoms and can lead to impaired activities of daily living (ADL) and a lower quality of life [2,3]. Recently, some novel rehabilitative interventions for hemiparesis after stroke were developed [4,5,6,7]. However, in the process of rehabilitation after stroke, it is still necessary to predict functional outcomes in order to set feasible goals and devise plans for daily living in the chronic phase [8,9]. If the functional outcomes after stroke could be predicted based on some neurophysiological investigations such as electroencephalography (EEG), devising appropriate plans for stroke rehabilitation would be easier. Especially, if the damage to neural networks in the brain could be evaluated with such investigations non-invasively in the early phase of stroke, it would be an important biomarker for the prediction of functional outcomes after stroke. 

On the other hand, some researchers clinically reported that the neural connections within the brain can be evaluated with EEG-based analysis to obtain the correlation coefficient [10,11,12]. Analysis to obtain the correlation coefficient can provide information about the similarity, relative amplitudes, and time delays between two EEG waveforms recorded in different cortical areas. The correlation coefficient can be considered as a quantitative measure of the degree of functional connectivity between distinct cerebral cortical regions. However, there is no information about how the correlation coefficient in some cortico-cortical areas can be modified by motor task activation. Furthermore, to date, the clinical importance of the change in the correlation coefficient with a given task has not been investigated in any neurological disease. If we could identify a correlation between the extent of the correlation coefficient change with motor task activation in the early stage of stroke and the functional outcome in the chronic phase, it would be very helpful for stroke rehabilitation. 

Therefore, the purpose of this study was firstly to investigate how the correlation coefficient in cortico-cortical areas of the brain changes with motor task activation and secondly to assess the usefulness of the correlation coefficient change with the activation to predict the motor functional outcomes of hemiparetic limbs after stroke.

## 2. Subjects and Methods

### 2.1. Study Subjects

Sixteen post-stroke hemiparetic patients were enrolled as the subjects of this study. They comprised consecutive patients who were admitted to our rehabilitation ward (Department of Rehabilitation Medicine, IUHW Ichikawa Hospital, Chiba, Japan) between July 2021 and July 2022, in order to receive long-term inpatient rehabilitation for several weeks. The inclusion criteria for this study were the following: (1) clinical diagnosis of stroke (cerebral infarction or intracerebral hemorrhage) with hemiparesis; (2) confirmation of stroke lesion location in the subcortical area of the cerebrum (no involvement of cerebral cortex) by brain CT/MRI; (3) Brunnstrom recovery stage (BRS) 2-5 for hand–fingers on the hemiparetic side [13]; (4) age on admission to our rehabilitation ward between 40 and 90 years; (5) time between stroke onset and admission to our rehabilitation ward of fewer than 50 days; (6) history of a single stroke only (no bilateral stroke lesions); (7) no consciousness disturbance on admission (ability, at least, to respond to verbal commands promptly); (8) no cognitive impairment on admission (Mini-Mental Examination Score of more than 20 points on admission); (9) no aphasic symptom (ability to communicate with others verbally, without any difficulty); (10) no active physical or mental illness requiring medical management on admission; (11) no past history of seizure; (12) no medication of anti-epileptic agents.

During hospitalization in our rehabilitation ward, a maximum of 180 min of rehabilitative training was provided daily, based on the Japanese insurance system for patients in a rehabilitation ward (6 days/week) [14]. If the main symptom is hemiparesis after stroke, for example, 2 sessions of 60-min physical therapy such as gait training, and 1 session of 60-min occupational therapy such as ADL training were provided daily. When the general condition of the patient was unstable (e.g., slight fever, excessive fatigue, and local pain), the duration of rehabilitative training was shortened. All rehabilitative training was provided as one-to-one training by certificated rehabilitation therapists.

### 2.2. Evaluation of Motor Function

The motor function of hemiparetic upper and lower limbs was serially evaluated on the day of admission to our hospital and 4 weeks after the admission. For the evaluation, the Fugl–Meyer Assessment (FMA) was administered by a physical therapist and an occupational therapist in our department. The FMA is a performance-based quantitative measure for the assessment of various impairments in post-stroke patients [15]. The evaluation includes the measurement of voluntary movement, velocity, coordination, and reflex activity. This domain has a total of 100 points for a normal motor function. The maximum scores for a normal motor function are 66 and 34 points for the upper and lower limb, respectively.

### 2.3. EEG Recording at Rest and during Motor Task, and Data Preprocessing

EEG recording was performed within 5 days of the admission to our rehabilitation ward. EEG signals were recorded using digital EEG-1260 (NIHON KOHDEN, Japan) with Ag/AgCl electrodes. Nineteen EEG electrodes (Fp1, Fp2, F7, F8, F3, F4, Fz, T3, T4, C3, C4, Cz, T5, T6, P3, P4, Pz, O1, and O2) were placed according to the international 10–20 EEG system. The impedance of electrodes was calibrated under 10 kΩ. The low- and high-pass filters for the EEG recordings were 0.5 and 60 Hz, respectively.

**Figure 1:**
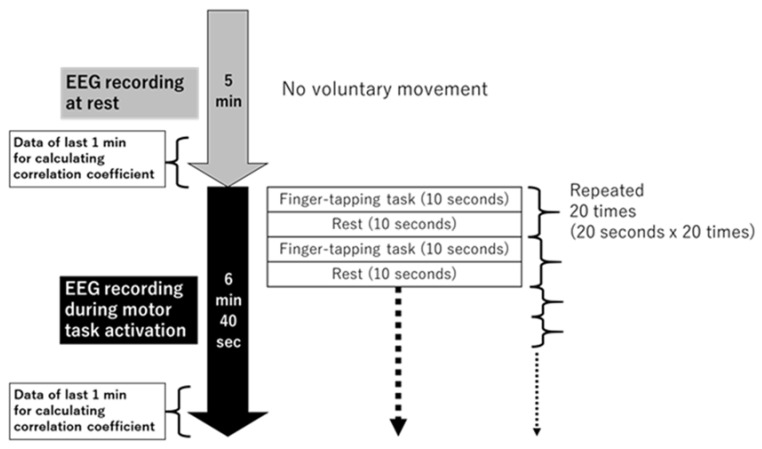
Protocol of motor task activation for studied patients.

EEG recording was performed at rest and during motor task activation, in order to assess how the correlation coefficient promptly changes with motor task activation. The subjects adopted a supine position on a comfortable bed for recording under both conditions. During EEG recording at rest, the subjects were asked to avoid unnecessary movements and keep their eyes closed. Following the 5-min recording at rest, a motor activation task was administered to the subjects for 6 min and 40 s (Figure 1). For motor task activation, the subjects were instructed to touch the thumb of the hemiparetic upper limb to the tips of their 2nd to 5th fingers on the same limb at a self-paced speed in turn repeatedly for 10 s, followed by 10-s rest. This 10-s finger-tapping task followed by 10-s rest was administered to the subjects 20 times. Some subjects were unable to touch their thumb to any other finger, although they could move their own fingers voluntarily. In such cases, the subjects still attempted to perform the task. An evaluator (F.Z. or W.K.) visually confirmed the presence of finger movement and the absence of upper-arm movement (to avoid the influence of upper-arm movement on the EEG waveform) during motor task activation, although the extent of movement differed among subjects. From the EEG recordings for 5 min at rest and 6 min and 40 s during motor task activation, the EEG data obtained during the last 1 min were used for correlation analysis. 

### 2.4. Correlation Coefficient Analysis Based on EEG Data

For subsequent preprocessing and analysis steps to obtain the correlation coefficient, FOCUS (NIHON KOHDEN, Japan) software was used. We exported the EEG data to FOCUS software for each patient. With the use of FOCUS software, a correlation function between a reference channel and all the displayed channels, including the reference channel can be computed. This means that the squared correlation coefficient r^2^ can be calculated between any electrode pairs. We consider that this correlation coefficient can represent the extent of effective neural connections between two cortical areas. The range of correlation coefficient is from 0% (no synchronization, which means no effective neural connection) to 100% (maximum synchronization, which means the largest neural connection). We calculated the correlation coefficient by shifting the reference to one channel using 30 data sampling points in both directions relative to the other channels. At each shifted point (total: 61 points), one correlation coefficient was computed. In the last process of the analysis, the squared correlation coefficients were displayed as waveforms in the correlation window of the software. The correlation coefficients were derived for 12 electrode pairs (C3-F3, C4-F4, C3-F7, C4-F8, F3-F7, F4-F8, C3-T3, C4-T4, F3-T3, F4-T4, F7-T3, and F8-T4) in this study.

### 2.5. Statistical Analysis

All statistical analyses were performed with SPSS version 23.0 (SPSS Inc., Chicago, IL, USA). The change in the correlation coefficient between the recordings at rest and during motor task activation was analyzed using a two-tailed paired Student’s *t*-test. The rate of the FMA score change for the upper and lower limbs was calculated by the FMA score on admission and 4 weeks after admission with the following formula: Rate of FMA score change = (FMA score at 4 weeks after admission—FMA score on admission) ÷ FMA score on admission × 100. Similarly, the rate of the correlation coefficient change in each cortico-cortical connection was calculated by the correlation coefficient at rest and during motor task activation, with the following formula: Rate of correlation coefficient change = (correlation coefficient during motor task activation—correlation coefficient at rest) ÷ correlation coefficient at rest × 100. The rate of the correlation coefficient change was compared between patients with moderate–severe upper-limb hemiparesis (BRS 2-4 for hand–fingers) and those with mild hemiparesis (BRS 5 for hand–fingers), using the Mann–Whitney U test. For the comparison between the FMA score on admission and 4 weeks after admission, a two-tailed paired Student’s *t*-test was used. In this study, the association between the rate of the correlation coefficient change and the rate of the FMA score change during hospitalization was statistically evaluated using Pearson’s correlation coefficients. A *p*-value less than 0.05 was considered significant.

## 3. Results

The clinical characteristics of the studied patients are presented in Table 1. The mean age on admission to our ward was 73.4 ± 9.6 years old. The period between stroke onset and admission to our ward ranged from 11 to 45 days (mean, 22.0 ± 9.4 days). Stroke was classified into cerebral infarction in 13 patients and intracerebral hemorrhage in 3 patients. The severity of upper-limb hemiparesis was moderate–severe in 6 patients and mild in 10 patients. The FMA score increase during hospitalization was significant for both the upper and lower limbs (all *p*-values < 0.05).

### 3.1. Change in Correlation Coefficient with Motor Task Activation in Each Cortico-Cortical Area

Table 2 shows the change in the correlation coefficient with motor task activation in each cortico-cortical area of the lesional and non-lesional hemispheres. In five areas of the lesional hemisphere (except for F7-T3 or F8-T4), the correlation coefficient showed a tendency to increase with motor task activation. Among them, the increase in the correlation coefficient in C3-F3 or C4-F4, C3-F7 or C4-F8, and F3-F7 or F4-F8 of the lesional hemisphere was significant (*p* < 0.05). On the other hand, the correlation coefficient in the non-lesional hemisphere did not show any significant change, although it exhibited a tendency to increase in all areas of the non-lesional hemisphere. Table 3 shows the rate of the correlation coefficient change with motor task activation in each cortico-cortical area. On comparing the rate of the correlation coefficient change between the moderate–severe and mild hemiparetic patients, the rate did not differ significantly between the two patient groups in any cortico-cortical area.

### 3.2. Correlation between Change in Correlation Coefficient and FMA Score Change

**Figure 2:**
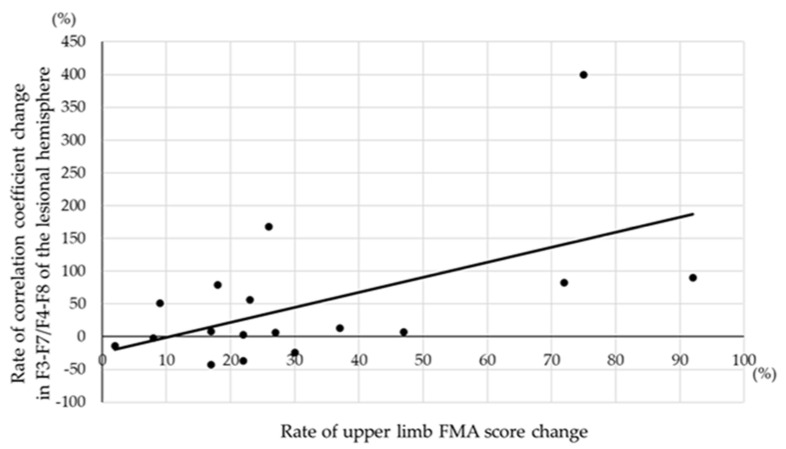
Scatter plots showing significant correlation between rate of correlation coefficient change in F3-F7/F4-F8 of the lesional hemisphere and rate of upper limb FMA score change.

Table 4 shows the correlation between the rate of change in the correlation coefficient with motor task activation and the rate of the FMA score change during 4 weeks after admission. The correlation was evaluated for both the upper- and lower-limb FMA scores. For the upper-limb FMA score, a significant positive correlation between the rate of the correlation coefficient change and the rate of the FMA score change was noted in F3-F7 or F4-F8 of the lesional hemisphere (r = 0.570, *p* < 0.05) (Figure 2). For the lower-limb FMA score, on the other hand, a significant correlation between these two rates was not identified in any cortico-cortical area. 

## 4. Discussion

Recently, some researchers applied correlation analysis based on EEG monitoring in order to evaluate the extent of neural functional connections in the brain of patients with neurological disorders, such as autism spectrum disorder, consciousness disturbance, and epilepsy [16,17,18]. Previously, we reported that the correlation coefficient in the lesional hemisphere significantly increased in association with motor functional recovery in post-stroke patients, indicating that neural connections in some cortico-cortical areas were augmented with rehabilitative training [19]. For the study, EEG monitoring to calculate the correlation coefficient was performed only at rest. We considered that the correlation coefficient more precisely reflects the functional connections between some cortical areas if EEG recording is performed during motor task activation in post-stroke hemiparetic patients. In addition, we also considered that the correlation coefficient during motor task activation more practically indicates how the neural connections related to the motor function are preserved after stroke, compared with the correlation coefficient at rest, in post-stroke patients. Therefore, in this study, we investigated the rate of the correlation coefficient change with motor task activation in post-stroke hemiparetic patients and identified a significant correlation between the rate in F3-F7 or F4-F8 of the lesional hemisphere and the motor functional recovery of the paretic upper limb. To the best of our knowledge, this is the first study to show that the extent of the EEG correlation coefficient change in the lesional frontal cortex can be used to predict the motor functional outcomes of post-stroke hemiparetic patients. 

With motor task activation, the correlation coefficient in some cortico-cortical areas of the lesional hemisphere significantly increased. In the convalescent phase of stroke, neural activity in the lesional hemisphere is generally increased on functional MRI scans to compensate for an impaired neurological function, although activation can be noted in the non-lesional hemisphere in the acute phase of stroke in some patients [20]. Similarly, Nelles et al. also showed that neural activity increases in association with motor functional recovery in the lesional hemisphere after the subacute phase of stroke [21]. Therefore, it is considered that our result of lesional neural activation with motor tasks indicates the possibility of the preservation of neural networks that can compensate for impaired neurological functions after stroke. In most of the studied stroke patients, the motor function significantly improved during their hospitalization, revealing that the neural function was successfully compensated for in some areas of the brain. 

In this study, we found that the extent of increases in the correlation coefficient in F3-F7 or F4-F8 of the lesional hemisphere was significantly correlated with improvement in the upper-limb motor function. This result means that the extent of the preservation of neural connections in the lesional prefrontal cortex can influence motor functional recovery after stroke. Previously, some researchers also reported that the extent of neural activation with certain motor tasks in the lesional prefrontal–frontal areas can be a predictor of motor functional outcome after stroke. Applying functional MRI with motor tasks, Loubinoux et al. demonstrated that motor functional recovery is more facilitated if higher neural activation is identified in the lesional prefrontal cortex in stroke patients [22]. In addition, with the results of a meta-analysis based on 24 functional neuroimaging studies, Fabre et al. showed that the extent of neural activation with the task in the lesional prefrontal cortex can be used to predict motor functional recovery [23]. The present findings are consistent with these previous studies. If the correlation coefficient is increased with motor task activation in F3-F7 or F4-F8 of the lesional hemisphere, this means that the functional connection in the lesional prefrontal cortex is mildly damaged but still preserved after stroke. In motor execution and motor imagery, some cortico-cortical networks between motor and cognitive areas are activated [24]. As Sharma et al. reported, the modulation of extant motor networks is needed for motor functional recovery after stroke, and the augmentation of neural connectivity in the lesional prefrontal cortex can improve the planning of movement, leading to the recovery of hemiparesis [25,26]. Therefore, it is considered clinically important to evaluate the change in the correlation coefficient with motor tasks in F3-F7 or F4-F8 of the lesional hemisphere in order to predict the response to the rehabilitative training of stroke patients. Importantly, this is the first study to successfully evaluate the extent of the damage to neural connections in the lesional prefrontal cortex with EEG recording, instead of neuroimaging studies for stroke patients. 

The present study had certain limitations. First, the number of the studied patients was relatively small. Some other clinical factors such as age at stroke onset, type of stroke, and the size and location of the stroke lesion can influence the EEG correlation coefficient change with motor activation task. In this study, we were not able to investigate the influence of these clinical factors on the change in the EEG correlation coefficient, because of the limited sample size of this study. Second, EEG recording to assess the correlation coefficient change with motor task activation was applied only on admission to our ward. No follow-up EEG monitoring was performed on any patient. It may be informative to compare the correlation coefficient change with a specific task between admission and follow-up, such as on discharge. Third, for the studied subjects, EEG recording was applied in their subacute phase. In this study, no subject undertook EEG recording in the acute phase, such as within 7 days of stroke onset. If we can identify a correlation between the response of the correlation coefficient with motor task activation in the acute phase and motor functional outcome, the prediction of the outcome could be possible earlier in the process of stroke rehabilitation.

## 5. Conclusions

With motor task activation, a significant increase in the EEG correlation coefficient was noted in some cortico-cortical areas of the lesional hemisphere in post-stroke hemiparetic patients. In particular, the increase in the correlation coefficient in F3-F7 or F4-F8 of the lesional hemisphere was significantly correlated with the increase in the upper-limb FMA score during 4 weeks after admission. It is considered that the increase in the EEG correlation coefficient with motor task activation in F3-F7 or F4-F8 of the lesional hemisphere can be a predictor of the motor functional outcomes of hemiparetic upper limbs after stroke. If we can predict the motor functional outcomes of stroke patients with the use of EEG recording, it may be possible to provide a rehabilitative program that is more suitable for each patient, leading to better functional outcomes after stroke. Undoubtedly, EEG recording is a non-invasive investigation for stroke patients and is available in most general hospitals. Therefore, our proposed method for the prediction of motor functional outcomes with the EEG correlation coefficient change can be introduced at many general hospitals. Further studies are needed to confirm the clinical usefulness of measuring the EEG correlation coefficient change with motor task activation in post-stroke hemiparetic patients. 

## Figures and Tables

**Table 1:** Clinical characteristics of studied patients (n = 16).

Age at admission to our ward, years old		73.4 ± 9.6
Gender		Female: 5 Male: 11
Diagnosis		Cerebral infarction: 13 Intracerebral hemorrhage: 3
Period between stroke onset and admission, days		22.0 ± 9.4
Side of legion (side of hemiparesis)		Left cerebral hemisphere (right hemiparesis): 8 Right cerebral hemisphere (left hemiparesis): 8
Brunnstrom Recovery Stage for hand–fingers at admission		Stage II: 3 Stage III: 0 Stage IV: 3 Stage V: 10
Period between admission and EEG, days		3.9 ± 2.0
Length of hospitalization, days		68.1 ± 26.5
FMA score at admission, points	Upper limb	41.2 ± 18.2
	Lower limb	21.8 ± 7.5
FMA score 4 weeks after admission, points	Upper limb	51.6 ± 19.5
	Lower limb	27.3 ± 7.3

**Table 2:** Changes in correlation coefficient with motor task activation.

	Measured Areas	Correlation Coefficient at Rest, %	Correlation Coefficient during Motor Task Activation, %	*p*-Value
Within lesional hemisphere	C3-F3 or C4-F4	18.9 ± 15.0	31.1 ± 22.6	<0.05
	C3-F7 or C4-F8	17.1 ± 15.0	27.6 ± 20.4	<0.05
	F3-F7 or F4-F8	45.2 ± 20.5	60.6 ± 17.6	<0.05
	C3-T3 or C4-T4	27.4 ± 16.7	35.6 ± 17.5	0.061
	F3-T3 or F4-T4	14.8 ± 14.5	27.9 ± 22.7	0.075
	F7-T3 or F8-T4	42.9 ± 13.3	41.1 ± 17.4	0.767
Within non-lesional hemisphere	C3-F3 or C4-F4	25.3 ± 19.8	31.4 ± 20.3	0.191
	C3-F7 or C4-F8	20.1 ± 14.3	25.5 ± 17.9	0.364
	F3-F7 or F4-F8	48.4 ± 14.2	50.4 ± 19.3	0.730
	C3-T3 or C4-T4	27.3 ± 17.5	32.5 ± 20.6	0.393
	F3-T3 or F4-T4	17.2 ± 18.6	19.6 ± 16.9	0.731
	F7-T3 or F8-T4	37.9 ± 12.2	38.8 ± 21.7	0.893

**Table 3:** Rate of correlation coefficient change with motor task activation.

	Measured Areas	All Patients (n = 16)	Moderate–Severe Hemiparetic Patients (n = 6)	Mild Hemiparetic Patients (n = 10)	*p*-Value between Moderate–Severe and Mild Patients
Within lesional hemisphere	C3-F3 or C4-F4	21.4 (−16.0–45.5)	−17.1 (−55.9–149.2)	10.8 (−24.7–29.4)	0.713
	C3-F7 or C4-F8	13.5 (−23.5–55.4)	0.0 (−83.9–205.6)	27.8 (−8.3–90.8)	0.181
	F3-F7 or F4-F8	7.3 (−11.1–81.6)	82.4 (31.4–245.1)	0.8 (−16.7–6.2)	0.181
	C3-T3 or C4-T4	4.0 (−13.6–38.7)	4.0 (−40.0–255.0)	27.9 (7.3–49.2)	0.792
	F3-T3 or F4-T4	42.9 (4.8–100)	27.3 (−31.0–116.7)	52.7 (−2.8–103.6)	0.864
	F7-T3 or F8-T4	−4.4 (−27.1–22.7)	−20.8 (−60.2–16.6)	2.8 (−10.7–30.4)	0.428
Within non-lesional hemisphere	C3-F3 or C4-F4	1.3 (−27.7–18.0)	−25.0 (−34.1–114.1)	1.3 (−26.5–10.6)	0.875
	C3-F7 or C4-F8	0.0 (−44.0–47.3)	0.0 (−60.9–0.0)	40.1 (−14.4–101.3)	0.108
	F3-F7 or F4-F8	5.1 (−33.2–37.8)	37.8 (−16.1–105.9)	17.6 (−26.7–47.2)	0.263
	C3-T3 or C4-T4	0.8 (−32.9–25.7)	−12.8 (−45.7–26.7)	10.5 (−26.0–44.3)	0.492
	F3-T3 or F4-T4	−24.2 (−68.1–58.9)	4.8 (−48.3–68.6)	−30.9 (−75.0–76.8)	0.298
	F7-T3 or F8-T4	−18.9 (−43.4–11.1)	−21.8 (−67.4–0.8)	−25.1 (−47.4–−1.0)	0.792

Moderate–severe hemiparesis: Brunnstrom recovery stage 2-4 for hand–fingers; Mild hemiparesis: Brunnstrom recovery stage 5 for hand–fingers. The data were presented as median (Interquartile Range: IQR).

**Table 4:** Correlation between correlation coefficient change and FMA score change.

		Upper-Limb FMA Score		Lower-Limb FMA Score	
		Coefficient	*p*-Value	Coefficient	*p*-Value
Within lesional hemisphere	C3-F3 or C4-F4	0.209	0.483	−0.111	0.682
	C3-F7 or C4-F8	−0.004	0.987	−0.332	0.208
	F3-F7 or F4-F8	0.570	<0.05	0.036	0.894
	C3-T3 or C4-T4	0.381	0.145	0.039	0.887
	F3-T3 or F4-T4	−0.068	0.804	−0.251	0.349
	F7-T3 or F8-T4	−0.231	0.389	−0.421	0.104
Within non-lesional hemisphere	C3-F3 or C4-F4	0.212	0.430	0.202	0.452
	C3-F7 or C4-F8	−0.238	0.374	−0.141	0.602
	F3-F7 or F4-F8	0.343	0.193	0.025	0.927
	C3-T3 or C4-T4	−0.066	0.809	0.180	0.506
	F3-T3 or F4-T4	−0.098	0.718	−0.067	0.804
	F7-T3 or F8-T4	−0.134	0.620	−0.047	0.862

## Data Availability

All data generated or analyzed during this study are included in this article. Further enquiries can be directed to the corresponding author.
